# Simulating Synaptic Behaviors through Frequency Modulation in a Capacitor–Memristor Circuit

**DOI:** 10.3390/mi14112014

**Published:** 2023-10-29

**Authors:** Kuibo Yin, Jingcang Li, Yuwei Xiong, Mingyun Zhu, Zhiyuan Tan, Zhanrui Jin

**Affiliations:** SEU-FEI Nano-Pico Center, Key Laboratory of MEMS of Ministry of Education, Southeast University, Nanjing 210096, China

**Keywords:** memristors, LTD, LTP, synaptic plasticity, Hebbian-like learning mechanism

## Abstract

Memristors, known for their adjustable and non-volatile resistance, offer a promising avenue for emulating synapses. However, achieving pulse frequency-dependent synaptic plasticity in memristors or memristive systems necessitates further exploration. In this study, we present a novel approach to modulate the conductance of a memristor in a capacitor–memristor circuit by finely tuning the frequency of input pulses. Our experimental results demonstrate that these phenomena align with the long-term depression (LTD) and long-term potentiation (LTP) observed in synapses, which are induced by the frequency of action potentials. Additionally, we successfully implement a Hebbian-like learning mechanism in a simple circuit that connects a pair of memristors to a capacitor, resulting in observed associative memory formation and forgetting processes. Our findings highlight the potential of capacitor–memristor circuits in faithfully replicating the frequency-dependent behavior of synapses, thereby offering a valuable contribution to the development of brain-inspired neural networks.

## 1. Introduction

While the brain lags behind computers in terms of computational speed and accuracy, it possesses a remarkable advantage in its ability to perform massively parallel processing [1,2,3]. This advantage stems from the brain’s vast number of neurons and synapses, as well as its ability to process analog signals [1,3,4]. As the connections between neurons, synapses play a crucial role in this process [5,6,7]. The plasticity of synapses, including long-term potentiation (LTP) and long-term depression (LTD) [8,9], is considered to be the biological basis of learning and memory [4,6]. The development of electronic devices capable of emulating the behavior of biological synapses is considered fundamental to the construction of brain-inspired neural networks [10].

The concept of the memristor was first proposed by Chua in 1971 to describe the relationship between flux-linkage and charge. It was subsequently developed and produced by HP in 2008 [11,12]. The adjustable conductance of the memristor bears similarity to the weight of synapses [5,6,13]. However, the conventional method of modifying memristor conductance using positive and negative voltages fails to account for the influence of action potential frequency on synaptic weight changes [5]. In biological synapses, high-frequency stimulation leads to LTP, while low-frequency stimulation induces LTD [14]. Previous attempts to introduce frequency dependence in conductance modification, such as using diffusion memristors and second-order drift memristors, have yielded results that deviate from synaptic behavior [15,16]. Therefore, the challenge of accurately mimicking the frequency-dependent behavior of synapses remains unresolved.

In this study, we propose a capacitor–memristor circuit as a means of emulating the frequency-dependent behavior of biological synapses. We first investigate the mechanism of the capacitor–memristor circuit by applying rectangular pulses as an input. Our findings reveal the emergence of positive or negative voltage spikes across the memristor on the rising or falling edges of the input rectangular pulse, respectively. We then introduce specialized pulses, called action pulses, into the capacitor–memristor circuit to emulate synaptic LTP and LTD. High-frequency pulses result in an increase in memristor conductance, akin to LTP, while low-frequency pulses lead to a decrease in conductance, similar to LTD in synapses. Finally, by connecting two memristors to the same capacitor, we achieve a Hebbian-like learning mechanism, where the increase and decrease in conductance signify the processes of associative memory formation and forgetting, respectively.

## 2. The Comprehensive Physical Model of an Oxide Memristor

In the simulation, we utilized a tantalum oxide-based memristor model, which has been validated to closely match experimental results [17,18]. This model incorporates multi-physical field coupling and is solved using COMSOL.

The equations describing the drift and diffusion of oxygen vacancy (*V_O_*) concentration (*n_D_*) are represented by Equations (1) and (2), respectively. [17,18]. *D*∇*n_D_*, *vn_D_* and *DSn_D_*∇*T* are the Fick diffusion flux, drift flux, and Soret diffusion flux, respectively. *f* is the escape attempt frequency (10^12^ Hz), a is the hopping distance (0.1 nm), and *E_a_* is the diffusion barrier (0.85 eV).

(1) $$\frac{\partial n_{D}}{\partial t}=\nabla \cdot (D\nabla n_{D}-vn_{D}+DSn_{D}\nabla T)$$

(2) $$\left\{\begin{array}{c} D=\frac{1}{2}\cdot a^{2}\cdot f\cdot \exp\left(-E_{a}/k/T\right)\\ v=a\cdot f\cdot \exp\left(-E_{a}/k/T\right)\cdot \sinh\left(qaE/k/T\right)\\ S=-E_{a}/\left(KT^{2}\right) \end{array}\right.$$

The current continuity equation and Joule heating equation are Equations (3) and (4), respectively. *σ* and *k_th_* are the electrical conductivity and thermal conductivity, respectively [17,18].

(3) ∇·σ∇Ψ=0

(4) $$-\nabla \cdot k_{th}\nabla T=J\cdot E$$

**Figure 1:**
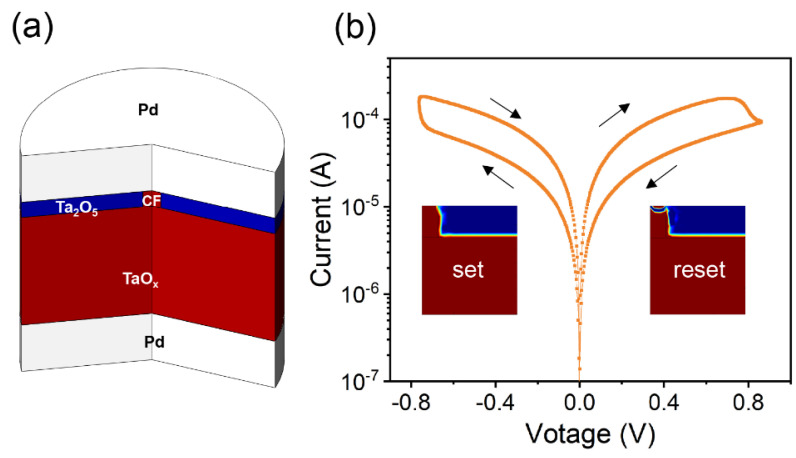
Modeling a tantalum oxide memristor. (**a**) The schematic of the tantalum oxide-based memristor used in the simulation. (**b**) Calculated DC I-V characteristics of the Pd/TaO_x_/Ta_2_O_5_/Pd device.

Figure 1a illustrates the structure of the memristor, with the Ta_2_O_5_ film serving as the resistive switching (RS) layer and the TaO_x_ film acting as the *V_O_* reservoir. Both the top and bottom electrodes are made of Pd. Figure 1b depicts a sweep voltage curve, clearly showing the set and reset processes. The arrows in the Figure 1b indicate the sweeping direction of voltage.

## 3. The Capacitor–Memristor Circuit with Rectangular Pulses as the Input

**Figure 2:**
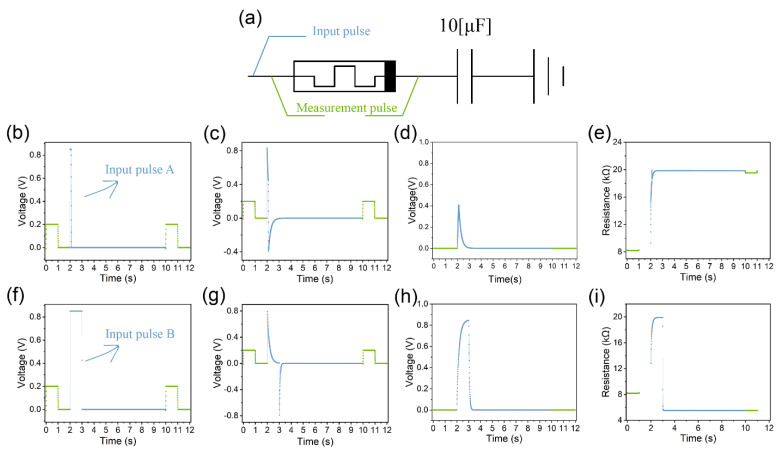
The simulation results of rectangular pulses as the input. (**a**) The capacitor–memristor circuit used for the simulation. (**b**) For the input pulse A (blue) and measurement pulses (green), the measurement pulses are applied directly across the memristor to measure the resistance of the memristor, as shown in subfigure (**a**). (**c**) The voltage across the memristor when the rectangular pulse A is used as the input. (**d**) The voltage across the capacitor when the rectangular pulse A is used as the input. (**e**) The resistance of the memristor when the rectangular pulse A is used as the input. (**f**–**i**) The corresponding results of the input pulse B.

Figure 2a depicts a capacitor–memristor circuit and illustrates the application method of the input and measurement pulses. In Figure 2b, the blue curve represents the input pulse A, and the green curve is the measurement pulse.

It is evident from Figure 2c that a positive voltage spike occurs across the memristor at the rising edge of the input pulse A. This phenomenon can be attributed to the rapid increase in voltage of the input pulse A, causing most of the voltage to be applied across the memristor due to the limited ability of the capacitor voltage to change abruptly. Subsequently, as depicted in Figure 2d, the voltage across the memristor decreases while the voltage across the capacitor increases. The rate of increase in the capacitor voltage is determined by the time constant *τ* (*τ* = *R∙C*), with a smaller time constant resulting in a faster increase in the capacitor voltage.

When the voltage of the input pulse A drops to zero, the capacitor discharges through the memristor, leading to a negative voltage spike across the memristor, as shown in Figure 2c. The magnitude of this negative voltage spike depends on the amount of charge accumulated in the capacitor. A wider input pulse (Figure 2f) causes a greater charge accumulation in the capacitor (Figure 2h), consequently resulting in a more pronounced negative voltage spike (Figure 2g).

Positive and negative voltage spikes induce an increase and decrease in the memristor resistance, respectively. The final resistance state is determined by the interplay between these two spikes (Figure 2e,i). Our simulation results are in line with the experimental findings of Kim et al. [19].

## 4. The Theoretical Analysis of the Capacitor–Memristor Circuit

### 4.1. The Rectangular Pulse as the Input

**Figure 3:**
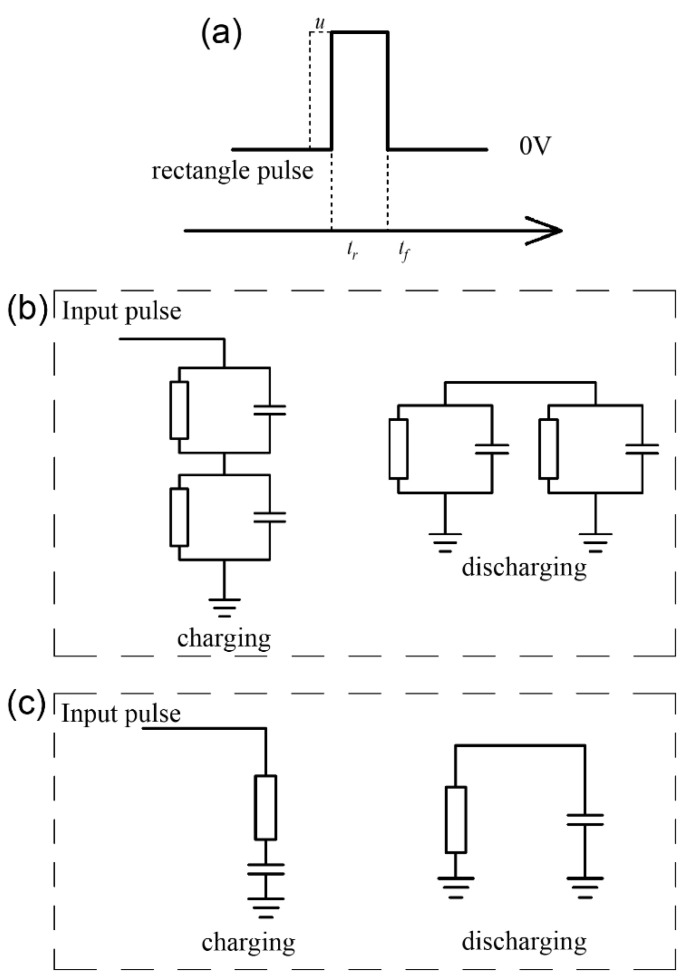
The working mechanism of the capacitor–memristor circuit. (**a**) Rectangular pulse. (**b**) A schematic diagram of the charging and discharging of the capacitor when a rectangular pulse is applied. (**c**) Simplified diagram of the charging and discharging of the capacitor when a rectangular pulse is applied.

To calculate the voltage across the memristor when a rectangular pulse (Figure 3a) is applied, we consider the memristor as a resistance *R_m_* in parallel with a parasitic capacitance *C_m_*. The capacitor is considered as a capacitance *C_c_* in parallel with a leakage resistance *R_c_*, as shown in Figure 3b [20,21]. To simplify the analysis, the resistance of the memristor is assumed to be constant. The time constant of this circuit can be determined from Equation (5) [22] as follows:

(5) $$\tau =\frac{R_{m}R_{c}}{R_{m}+R_{c}}\cdot \left(C_{m}+C_{c}\right)$$

The rising edge of the rectangular pulse is at *t_r_*, and the falling edge is at *t_f_*. It is assumed that there is no charge accumulation in the capacitor before *t_r_*. The voltage across the memristor is as follows:

(6) $$\left\{\begin{array}{c} u_{m}=u\cdot \left(\frac{C_{c}}{C_{m}+C_{c}}-\frac{R_{m}}{R_{m}+R_{c}}\right)\cdot e^{-\frac{t-t_{r}}{\tau }}+\frac{R_{m}}{R_{m}+R_{c}}\cdot u,\;\;t_{r}<t<t_{f}\;\;\;\\ u_{m}=-\left(\frac{C_{c}u}{C_{c}+C_{m}}-u_{mf}\right)\cdot e^{-\frac{t-t_{f}}{\tau }},\;t_{f}<t \end{array}\right.$$

where u is the amplitude of the rectangular pulse, and u_mf_ is the voltage across the memristor at $t\to t_{f}^{-}$. In general, the parameters in Equations (5) and (6) satisfy *C_c_* ≫ *C_m_* and *R_c_* ≫ *R_m_* [21,23]. Thus, the circuit in Figure 3b can be simplified to the circuit in Figure 3c. Equations (5) and (6) can be simplified to

(7) $$\tau =R_{m}\cdot C_{c}$$

(8) $$\left\{\begin{array}{c} u_{m}=u\cdot e^{-\frac{t-t_{r}}{\tau }}\;,\;\;\;\;\;\;\;t_{r}<t<t_{f}\;\;\\ u_{m}=-u_{cf}\cdot e^{-\frac{t-t_{f}}{\tau }}\;,\;\;\;\;\;\;\;t_{f}<t\;\;\;\; \end{array}\right.$$

(9) $$u_{cf}=u\cdot \left(1-e^{-\frac{t_{f}-t_{r}}{\tau }}\right)$$

where *u_cf_* is the voltage across the capacitor at $t\to t_{f}^{-}$. According to Equation (8), the positive voltage spike across the memristor is close to *u* regardless of the width of the rectangular pulse. However, the negative voltage spike across the memristor depends on the width of the rectangular pulse and the time constant. A wider pulse and a smaller time constant will result in a higher voltage across the memristor during discharge.

### 4.2. The Action Pulse as the Input

**Figure 4:**
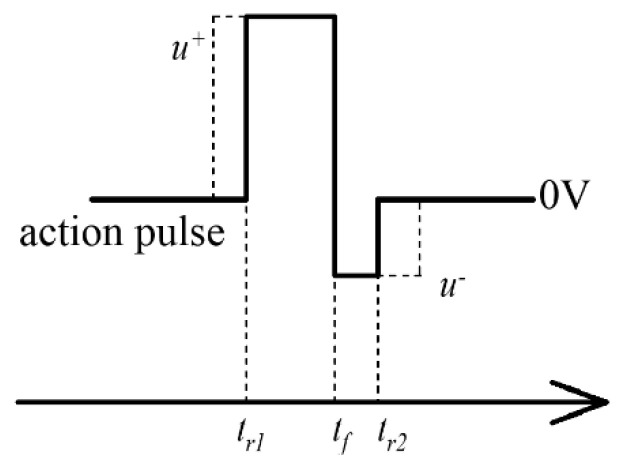
The action pulse is a combination of a positive rectangular pulse and a negative rectangular pulse.

From the above analysis, we can see that the resistance of the memristor can be modified during the discharge of the capacitor in the memristor–capacitor circuit. We then designed a special pulse called an action pulse (Figure 4). The action pulse is a combination of a positive rectangular pulse and a negative rectangular pulse. The negative rectangular pulse is used here to increase the voltage during discharge. It is assumed that there is no charge accumulation in the capacitor before *t_r1_*. The voltage across the memristor is

(10) $$\left\{\begin{array}{c} u_{m}=u^{+}\cdot e^{-\frac{t-t_{r1}}{\tau }}\;,\;t_{r1}<t<t_{f}\;\;\;\;\;\;\;\;\;\;\;\;\;\;\;\;\;\;\;\;\;\\ u_{m}=-(u_{cf}-u^{-})\cdot e^{-\frac{t-t_{f}}{\tau }}\;,\;t_{f}<t<t_{r2}\;\;\;\;\\ \;u_{m}=-u_{cr2}\cdot e^{-\frac{t-t_{r2}}{\tau }}\;,\;t_{r2}<t\;\;\;\;\;\;\;\;\;\;\;\;\;\;\;\;\;\;\;\;\;\;\;\;\; \end{array}\right.$$

(11) $$\left\{\begin{array}{c} u_{cf}=u^{+}\cdot \left(1-e^{-\frac{t_{f}-t_{r}}{\tau }}\right)\;\;\;\;\;\;\;\;\;\;\;\;\;\;\;\;\;\;\;\;\;\;\;\;\;\;\;\;\;\;\;\;\\ u_{cr2}=(u_{cf}-u^{-})\cdot e^{-\frac{t_{r2}-t_{f}}{\tau }}+u^{-}\;\;\;\;\;\;\;\;\;\;\;\;\;\;\; \end{array}\right.$$

where *u_cf_* is the voltage across the capacitor at $t\to t_{f}^{-}$, and *u_cr2_* is the voltage across the capacitor at $t\to t_{cr2}^{-}$. The positive voltage spike across the memristor during charging is close to u^+^. The negative voltage spike during discharge depends on *u_cf_* and *u*^−^. The existence of the negative rectangular pulse is necessary to increase the voltage across the memristor during discharge of the capacitor. From the equation we can see that the decay rate of the voltage spike is negatively related to the time constant *τ*. Importantly, if multiple pulses are input into the capacitor–memristor circuit at very short intervals, the charge in the capacitor will remain at the beginning of the subsequent pulse input. The remaining charge will reduce the voltage across the memristor during charging and increase the voltage during discharge, which will eventually affect the change in conductance.

## 5. Imitation of Synaptic Behavior Based on the Capacitor–Memristor Circuit

### 5.1. Long-Term Depression (LTD)

**Figure 5:**
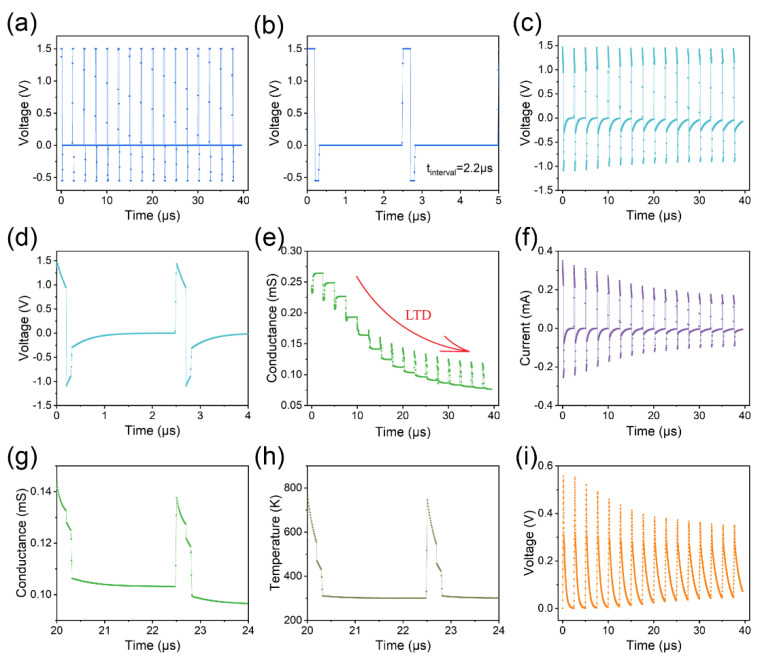
Input low-frequency action pulses to the capacitor–memristor circuit to emulate LTD. (**a**) Low-frequency action pulses with an interval of 2.2 μs. (**b**) The partial enlargement of subfigure (**a**). (**c**) The voltage across the memristor. (**d**) The partial enlargement of subfigure (**c**). (**e**) The conductance of the memristor. (**f**) The current through the memristor. (**g**) The conductance of the conducting filament. (**h**) The temperature of the conducting filament. (**i**) The voltage across the capacitor.

We employed action pulses in the capacitor–memristor circuit (with a capacitance value of 0.1 nF) to realize both LTD and LTP. To induce LTD, we input low-frequency action pulses into the circuit (Figure 5a,b). The positive voltage spikes across the memristor are close to 1.5 V, while the negative voltage spikes are around −1.1 V (Figure 5c,d). The interplay between these two spikes leads to a reduction in the memristor’s conductance (Figure 5e), as evidenced by the decrease in current depicted in Figure 5f. It should be noted that the fluctuations in memristor conductance are attributed to temperature variations. The conductivity of the conducting filament is assumed to follow the Arrhenius equation in the model $\sigma =\sigma_{0}\exp\left(-\frac{E_{AC}}{kT}\right)$, where *σ*_0_ is a pre-exponential factor, *k* is Boltzmann’s constant, and *E_AC_* is the activation energy for conduction [17,24,25]. As illustrated in Figure 5g,h, the conductance exhibits a positive correlation with temperature. The peak voltage drop across the capacitor can be attributed to the decrease in memristor conductance, which subsequently reduces the charging current (Figure 5i).

### 5.2. Long-Term Potentiation (LTP)

**Figure 6:**
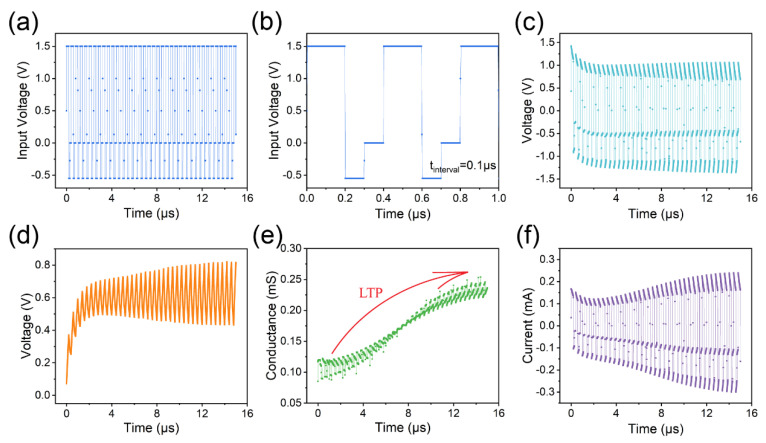
Input high-frequency action pulses to the capacitor–memristor circuit to emulate LTP. (**a**) High-frequency action pulse with an interval of 0.1 μs. (**b**) The partial enlargement of subfigure (**a**). (**c**) The voltage across the memristor. (**d**) The voltage across the capacitor. (**e**) The conductance of the memristor. (**f**) The current through the memristor.

To emulate LTP, we input high-frequency action pulses (Figure 6a,b) with an interval of 0.1 μs. As the number of input high-frequency pulses increases, the positive spike reduces to 1.0 V, while the negative spike approaches −1.3 V (Figure 6c). The charge within the capacitor remains when the next pulse arrives, and gradually accumulates with subsequent action pulses. The increase in capacitor voltage (Figure 6d) leads to a decrease in the voltage across the memristor during charging and an increase during discharging. A smaller capacitor (0.1 nF) is employed to allow a faster increase in capacitor voltage. Initially, the conductance of the memristor experiences a slight reduction with the first few pulses, but then increases as the number of input pulses rises (Figure 6e). The increase in current through the memristor also indicates an increase in conductance (Figure 6f).

### 5.3. A Hebbian-Like Learning Mechanism

**Figure 7:**
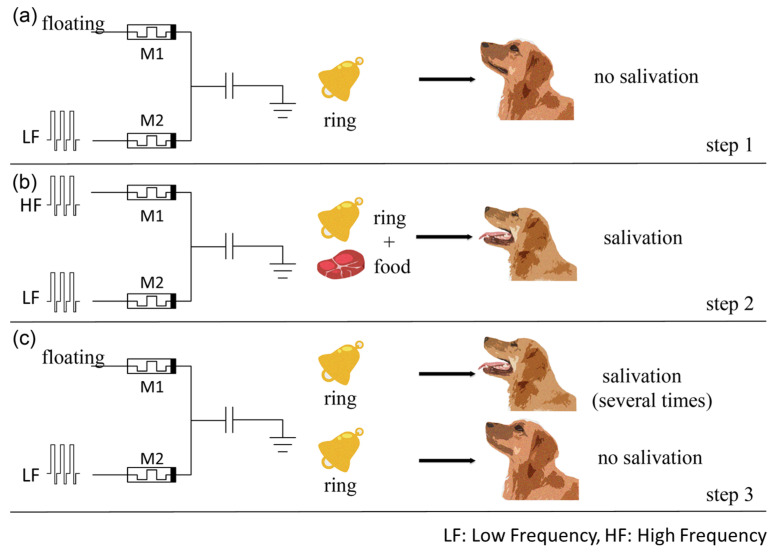
The implementation process of the Hebbian-like mechanism. (**a**) In the first step, the pulses are only input to memristor M2, while memristor M1 is floating. This step can be regarded as the dog not salivating when there is only the stimulation of the bell ring. (**b**) In the second step, high- and low-frequency pulses are input to memristors M1 and M2, respectively. During this step, the dog is stimulated by both the ring and the food at the same time. (**c**) The third step has the same input as the first step. The dog salivates the first few times when only the ring signal is given, but then is indifferent to the stimulus of the ring. These phenomena represent associative memory and forgetting.

Hebbian rules are widely recognized as the foundation of the learning and memory functions of neural networks, where the weight of the synapse is modified based on the correlated spikes of the pre- and post-synaptic neurons [26,27,28]. Many researchers have studied the Hebbian-like mechanism based on the SPICE model and achieved good results [29,30]. In this study, we used the COMSOL model of the memristor to construct a capacitor–memristor circuit to realize the Hebbian-like mechanism, as shown in Figure 7. The two pre-synaptic neurons are represented by voltage sources, which imitates the sight of food and the sound of a bell, respectively. Memristor M1 with a larger initial weight represents the synapse between the “food” pre-synaptic neuron and the “salivation” post-synaptic neuron, while memristor M2 with a smaller initial weight denotes the synapse connected to the “bell” pre-synaptic neuron [21]. The entire simulation process consists of three steps. In step 1 (Figure 7a), the dog does not salivate when only the bell signal is given. In step 2 (Figure 7b), the dog salivates when both the bell and food stimuli are applied simultaneously. This step is repeated several times. In step 3 (Figure 7c), the dog is exposed to the bell stimulus alone, similar to step 1. Initially, the dog salivates when the bell signal is given, indicating the acquisition of associative memory. However, after several repetitions of the bell stimulus, the dog no longer salivates, representing the forgetting process [31].

**Figure 8:**
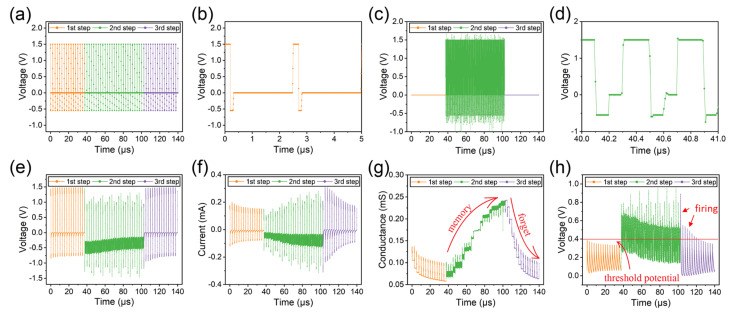
A Hebbian-like mechanism is implemented in the capacitor–memristors. (**a**) Input low-frequency action pulse to memristor M2. (**b**) The partial enlargement of subfigure (**a**). (**c**) Input high-frequency action pulse to memristor M1. (**d**) The partial enlargement of subfigure (**c**). (**e**) The voltage across memristor M2. (**f**) The current through memristor M2. (**g**) The conductance of memristor M2. (**h**) The voltage across the capacitor.

Figure 8 illustrates the results of simulating the Hebbian-like mechanism. Memristor M1 is replaced by a constant resistance (G = 0.25mS) to simplify the calculation. The input to memristor M1 is always a high-frequency pulse, while the input to the memristor M2 is a low-frequency pulse. In addition, we assume that the post-synaptic neuron fires when the voltage across the capacitor exceeds 0.4 V.

In step 1, the pulses are only applied to memristor M2, while memristor M1 is floating (yellow curves in Figure 8a–d). The conductance of memristor M2 decreases slightly due to the low-frequency pulses, similar to the LTD behavior (yellow curves in Figure 8e–g). The peak voltage across the capacitor decreases gradually and remains below 0.4 V (yellow curve in Figure 8h). Consequently, the firing of the “salivation” post-synaptic neuron is not triggered by the sound of the bell in step 1.

In step 2, we input appropriate pulses to memristors M1 and M2 simultaneously (green curves in Figure 8a–d). The high-frequency pulse input to M2 increases the voltage across the capacitor (green curve in Figure 8h), which causes the voltage spike across memristor M2 to decrease during charging and increase during discharging (green curve in Figure 8e). Finally, the conductance of memristor M2 increases as the number of input pulses increases (green curve in Figure 8g), which can be regarded as the process of associative memory.

In step 3, the input pulses to memristor M1 are removed, and the input pulses to memristor M2 are retained, as in step 1 (purple curves in Figure 8a–d). As a result, the conductance of the memristor gradually decreases as the number of input pulses increases (purple curve in Figure 8g). It is worth noting that the peak voltage across the capacitor exceeds 0.4 V during the first few pulses (purple curve in Figure 8h), which means that the “salivation” post-synaptic neuron is firing. In other words, it has learned in step 2 to salivate when only the ring signal is given. However, as the conductivity of the memristor continues to decrease, it gradually forgets this ability.

## 6. Conclusions

The capacitor–memristor circuit was simulated using COMSOL. With the well-designed action pulses, it was shown that the capacitor–memristor circuit can emulate the frequency-dependent behavior of synapses, in which high- and low-frequency pulses induce LTP and LTD, respectively. In addition, a Hebbian-like learning mechanism was realized by connecting two memristors to the same capacitor, which naturally shows the process of associative memory and forgetting.

## Data Availability

Data sharing not applicable.
